# Midterm Outcomes After Total Knee Arthroplasty With Lateral Approach for Valgus Knee Deformity in Patients With Rheumatoid Arthritis

**DOI:** 10.7759/cureus.58197

**Published:** 2024-04-13

**Authors:** Takaaki Noguchi, Makoto Hirao, Gensuke Okamura, Shigeyoshi Tsuiji, Jun Hashimoto

**Affiliations:** 1 Orthopaedic Surgery, National Hospital Organization, Osaka Minami Medical Center, Kawachinagano, JPN; 2 Orthopaedics and Rheumatology, Nippon Life Hospital, Osaka, JPN

**Keywords:** medial instability, lateral approach, total knee arthroplasty, valgus knee deformity, rheumatoid arthritis

## Abstract

Background

Valgus knee deformity is often seen in rheumatoid arthritis (RA) cases. Usually, the medial approach has been often utilized for total knee arthroplasty (TKA), even in valgus deformity cases; however, the medial approach is feared to induce further instability in the medial side because it could further break the soft tissue structure, including medial collateral ligament (MCL) and medial patellofemoral ligament (MPFL). Consequently, loosening of the implant, recurrence of valgus knee deformity, and pain due to instability might be induced in the early period after surgery. In this study, a lateral approach for TKA against valgus deformity in RA cases was utilized to avoid further damage on the medial side.

Methods

Eleven valgus knees in 10 patients with RA (mean age, 61.1 years; mean follow-up, 33.1 months) underwent primary TKA with the lateral approach. Iliotibial band (ITB) dissection and/or peroneal nerve release were performed if necessary. Radiological and clinical investigations were evaluated pre- and postoperatively.

Results

The average operating time was 106 minutes, which was no longer compared with the time after the medial approach described previously. The extension angle was significantly improved from −15.0 ± 10.2 to −5.5 ± 4.2 degrees (P = 0.03), while the flexion angle showed no significant change (from 111.8 ± 15.9 to 115.0 ± 13.2 degrees). The hip-knee-ankle angle (HKA) was also significantly corrected from −9 ± 4.9 to 0.4 ± 1.7 degrees (P < 0.001). The 2011 Knee Score System (KSS) scores were significantly improved from 6.9 ± 3.4 to 21.5 ± 2.9 (P < 0.001) in symptoms, from 15.6 ± 2.7 to 31.1 ± 4.1 (P < 0.001) in satisfaction, and from 31.5 to 59.5 (P < 0.01) in activity.

Conclusion

Midterm outcomes after lateral approach TKA were good, and knee alignment was significantly improved. The lateral approach TKA for valgus deformity in patients with RA was not complicated and difficult because it required no additional operating time compared with the medial approach. From the perspective of preventing further damage to the soft tissue structure on the medial side, the lateral approach was meaningful for valgus deformity in patients with RA.

## Introduction

With the progression of medical treatment for rheumatoid arthritis (RA), the frequency of total knee arthroplasty (TKA) in patients with RA cases has been decreasing in recent years [1]. However, valgus knee deformity is often seen in patients with RA, as compared with osteoarthritis (OA) [2]. Valgus knee is generally defined as femorotibial angle (FTA) ≤ 170 degrees [3] and characterized by contracture of soft tissues, such as iliotibial band (ITB), posterior cruciate ligament (PCL), lateral collateral ligament (LCL), and laxity of the medial collateral ligament (MCL), furthermore sometimes by hypoplasia of postero-lateral femoral condyle [4]. Even though these complexities of soft tissue and bony problems exist in valgus knee deformity, it is reported that only 10-15% of cases require the intervention of TKA [5,6]. Furthermore, TKA for valgus knee is generally performed with a medial approach [5,7]. However, the medial approach in TKA for the valgus knee is considered to be problematic from the perspective of maintaining the soft tissue balance because the medial approach could have the possibility to further damage the medial structure in addition to the situation of MCL laxity in valgus knee deformity. Excessive laxity of medial structures could also be a risk of impairment of patellar tracking [8] when the medial patellofemoral ligament (MPFL) has already been cut. In RA, soft tissue laxity could be induced easily due to pathological changes in collagenous tissue. Taken together, minimizing the release of medial soft tissue in the valgus knee deformity is desirable. Then, the lateral approach was considered meaningful and logical from the perspective of preventing damage to the medial structure. In this study, a lateral approach for TKA against valgus knee in patients with RA was evaluated.

## Materials and methods

Study design and patient population

Eleven consecutive cases with 10 RA patients suffering from end-stage valgus knee deformity were introduced in this study and retrospectively evaluated. All patients were treated with disease-modifying anti-rheumatic drugs (DMARDs), including methotrexate (MTX), and/or biologics to control RA disease activity. Patients who had undergone previous knee surgery were excluded. TKA with lateral approach was performed by one surgeon (T.N) from March 2017 to December 2021. The patients’ characteristics are shown in Table 1. This research was conducted in compliance with the Declaration of Helsinki and has been approved by the IRB of the National Hospital Organization, Osaka Minami Medical Center, Kawachinagano, Japan (approved number: R4-28). Informed consent has been obtained from all patients.

**Table 1:** Preoperative characteristics of patients with RA Data are presented as means ± SD unless otherwise noted. BMI, body mass index; DAS, Disease Activity Score; TCZ, tocilizumab; GLM, golimumab; ETN, etanercept

	N = 10
Age (y)	61.1± 17.0
male:female (n)	0:10
Disease duration (y)	25.8 ± 11.7
Weight (kg)	48.9 ± 8.2
BMI	18.9 ± 3.3
Steinbrocker stage (n)	
I	0
II	0
III	0
IV	10
Steinbrocker class (n)	
I	0
II	6
III	4
IV	0
DAS28-CRP score	2.97 ± 0.31
Prednisolone usage (%)	60.0
Prednisolone dosage (mg/day)	1.95 ± 2.50 (0 – 8)
Methotrexate usage (%)	70.0
Biologics usage (%)	50.0
Biologics (n)	
TCZ	1
GLM	3
ETN	1

Surgical procedure and postoperative procedure

**Figure 1:**
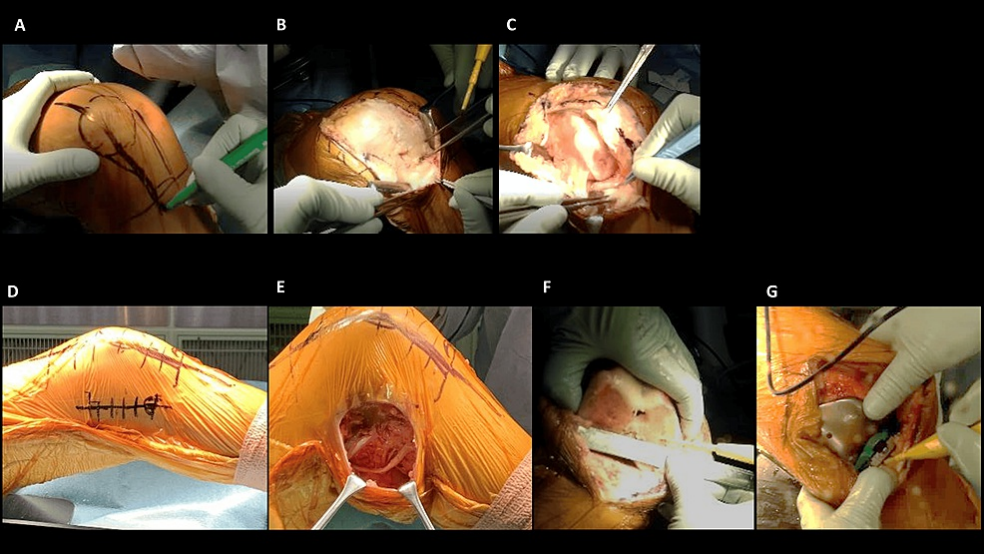
Skin incision and lateral approach for TKA (right knee) (A) A skin incision was placed on 1/3 outside the patella to the lateral tibial tubercle. (B) Dissecting is done just above the joint capsule and fascia. (C) Incision of the joint capsule using the lateral parapatellar approach to expose the knee joint. (D) Skin incision for peroneal nerve release (5 cm proximally from the fibular head). (E) Release of the peroneal nerve. (F) Determine the rotation of the femur by palpating the medial and lateral femoral condyles and drawing a line connecting them. (G) Determine tibial rotation using the ROM method. TKA, total knee arthroplasty; ROM, range of motion

TKA was performed using a lateral approach (Figures 1A-1C). ITB dissection was performed if the tightness of the lateral compartment was not fully released (Figures 1D, 1E). If flexion contracture of more than 30 degrees was added, peroneal nerve release was also added. A posterior-stabilized (PS) type implant was utilized in this study because PCL in valgus knee deformity generally tended to show contracture [7]. The patella was also replaced with polyethylene prostheses. Range of motion (ROM) exercise and full weight-bearing/gait exercise were started from day one after surgery. Suture removal was performed about two weeks after surgery.

Radiographic assessment

**Figure 2:**
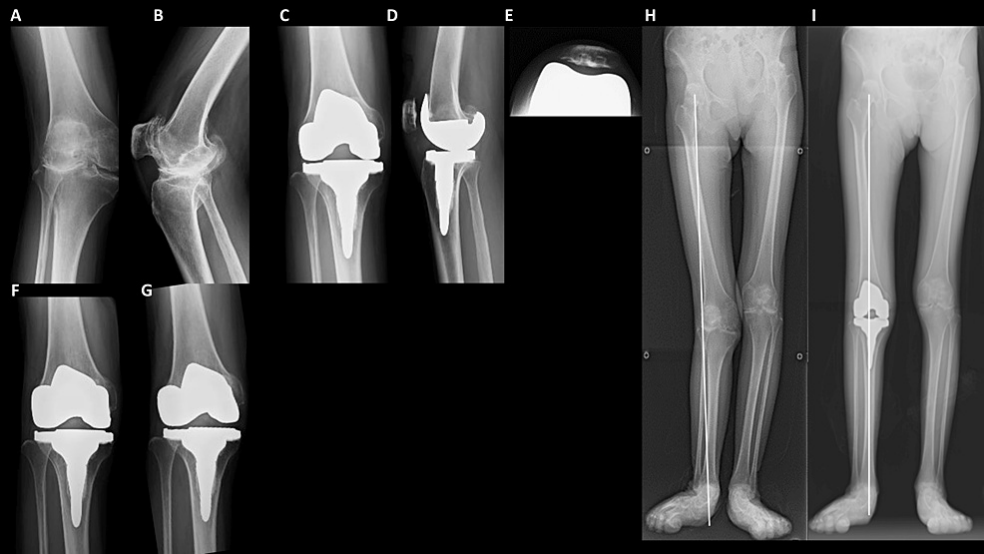
Radiographs of a representative case presentation A 48-year-old woman with a 22-year history of RA (Steinbrocker classification: stage IV class III) had a total 2011 KSS of 44 points (full score, 180 points). She could walk with a T cane for only a few minutes. (A) Preoperative radiograph of the anterior weight-bearing view. (B) Preoperative radiograph of the lateral weight-bearing view. (C) Postoperative radiograph of the anterior weight-bearing view. (D) Postoperative radiograph of the lateral weight-bearing view. (E) Postoperative radiograph of knee skyline view. The patellar position was good. (F) Antero-posterior view under varus stress. (G) Antero-posterior view under valgus stress. There was almost no instability under varus/valgus stressed condition. (H) Preoperative radiograph of the whole lower extremity. The white line shows the loading axis of the whole lower extremity. (I) Postoperative radiograph of the whole lower extremity. The passing point of the loading axis in the knee joint was centralized. RA, rheumatoid arthritis; KSS, Knee Score System

Weight-bearing radiographs of the knee were measured, and femoral coronal angle (FCA), tibial coronal angle (TCA), tibial sagittal angle (TSA), and hip-knee-ankle (HKA) angle were measured and evaluated [9,10]. A positive HKA angle indicates the varus alignment of the knee joint. Values of the alignment parameters in this study are shown in Table 2. Radiographic changes in a representative case are shown in Figure 2.

**Table 2:** Operation time for TKA and radiographic parameters of postoperative implantation Data are presented as means ± SD unless otherwise noted. TKA, total knee arthroplasty; FCA, femoral coronal angle; TCA, tibial coronal angle; TSA, tibial sagittal angle

Evaluation items	Measurement results
Operation time (minutes)	106.4 ± 22.5
FCA (°)	90.6 ± 1.7
TCA (°)	88.6 ± 1.2
TSA (°)	84.8 ± 1.7

Clinical assessment

Clinical findings and Knee Society Score (KSS) were recorded when patients did not face the surgeon at the time of preoperative and final follow-up (mean follow-up, 33.1 months). The timed up and go (TUG) test [11] was also performed at the preoperative and final follow-up.

Statistical analysis

All data are expressed as mean and standard deviation (SD) or median. Differences in measured variables between preoperative and postoperative scores were analyzed with the Mann-Whitney U test, as appropriate. Values of P < 0.05 were considered significant.

## Results

Intra- and postoperative events

Mean operating time was 106.3 minutes (range, 74-155). There was no case requiring both a constrained system and a hinge-type implant. ITB dissection was performed in five cases to release the contracture on the lateral side, and within these five cases, concomitant peroneal nerve release was performed in three cases. Deep infection was observed in one case eight months after surgery; however, fortunately, complete restoration was achieved without implant removal. The periprosthetic fracture was observed in one case 10 months after TKA by the fall, and then reduction and internal fixation were added; subsequently, the patient was able to walk without a cane.

Radiographic outcomes

After TKA, FCA was 90.6 ± 1.7 (88-93) degrees, TCA was 88.6 ± 1.2 (87-90) degrees, and TSA was 84.8 ± 1.7 (82-87) degrees. HKA alignment was significantly improved from −9.0 ± 4.9 to 0.4 ± 1.7 degrees (P < 0.001) (Table 2).

Clinical outcomes

Although it was not significant, the time of the TUG test was shortened from a mean of 15.02 to 12.31 seconds after surgery (Table 3). Mean knee extension was significantly improved from −15.0 to −5.5 degrees, while mean knee flection did not show significant change (from 111.8 to 115.0 degrees) (Table 3). In the 2011 KSS, symptoms, satisfaction, and activity significantly improved after TKA; meanwhile, there was no change in expectations before and after surgery (Table 4).

**Table 3:** ROM of the knee, radiographic knee alignment, and TUG time at preoperative and final follow-up time Data are presented as means ± SD unless otherwise noted. HKA, hip-knee-ankle angle; TUG, timed up and go; ROM, range of motion

	Preoperative	Final follow-up	P-value
Flexion (°)	111.8 ± 15.9	115.0 ± 13.2	0.67
Extension (°)	-15 ± 10.2	-5.5 ± 4.2	0.03
HKA (°)	-9 ± 4.9	0.4 ± 1.7	<0.001
TUG time (seconds)	15.0 ± 7.7	12.3 ± 5.6	0.15

**Table 4:** Changes in the KSS at preoperative and final follow-up time Data are presented as means ± SD unless otherwise noted. KSS, Knee Score System

	Preoperative	Final follow-up	P-value
Symptom (max: 25 points)	6.9 ± 3.4	21.5 ± 2.9	<0.001
Satisfaction (max: 40 points)	15.6 ± 2.7	31.1 ± 4.1	<0.001
Expectation (max: 15 points)	10.8 ± 2.5	9.9 ± 1.2	0.11
Activity (max: 100 points)	31.5 ± 21.3	59.5 ± 25.6	0.01

## Discussion

In our previous report, patients with RA and valgus knee deformity had relatively low physical activity because HKA had a negative correlation with TUG time [12]. Furthermore, if tight disease control by medication is not achieved, the valgus deformity has a possibility to progress due to degeneration of collagenous tissue (MCL) in the pathological duration of inflammatory RA disease and erosive/collapsing bone deformity. Therefore, knee reconstruction with varus correction should be recommended for patients with RA and valgus knee deformity to improve gait dysfunction. Once varus correction was achieved by TKA, soft tissue balance and such correction should be maintained permanently. However, when TKA for valgus knee deformity is performed with a medial approach, it is problematic to the point that not only further damage in medial side soft tissue, including MCL, might be induced, but also the release of the soft tissue in the lateral side of patella is difficult, subsequently inducing the impaired patellar tracking, due to relative laxity in the medial side [8], which might have a possibility to require early revision TKA due to pain and the appearance of knee instability [13]. Furthermore, there are cases where conventional implants are changed to constrained implants or rotating hinge knee (RHK) implants [14]. To avoid such situations, primary TKA with a lateral approach might be recommended from the perspective of not only preventing further soft tissue damage on the medial side but also making it easier to perform lateral release for controlling the patellar tracking. It is also easier to release the ITB as necessary in the same skin incision of the lateral approach. In valgus knee deformity, hypoplasia of the lateral femoral condyle is often seen, which makes it difficult to determine the rotation. It should be taken care to avoid malpositioning of the femoral component in abnormal rotation by direct confirmation of the medial and lateral epicondyle, as shown in Figure 1F. The lateral approach could make it easier to confirm the lateral epicondyle directly. On the other hand, concerning the rotation of the tibial component, it is difficult to see the medial side of the tibia in the lateral approach. Then, the rotational positioning of the tibial component was determined by the ROM method, as shown in Figure 1G. Even with the strong points mentioned above, the lateral approach is not considered complicated and difficult because it requires no additional operating time (current study, 106 minutes) as compared with the medial approach, as described previously (137 minutes) [14].

The limitation is that the current study was not a comparative study with a control group: medial approach. Furthermore, further longer follow-ups with an increased number of cases should be done in the future. Laxity of medial side soft tissue, including MCL, might have a possibility to progress because RA is one of the collagen diseases, and it is known that bare area, including the insertion of MCL and capsule, is a frequently occurring site of synovitis; subsequently, laxation of these soft tissues could be progressed in the long-term RA disease pathophysiology [15]. Then, tight control by novel medication after surgery and careful observation in the long-term follow-up also should be continued.

## Conclusions

Midterm outcomes after lateral approach TKA were good, and knee alignment was significantly improved. Furthermore, lateral approach TKA for valgus deformity in patients with RA was not complicated and difficult because it required no additional operating time as compared with the medial approach. From the perspective of preventing further damage to the soft tissue structure on the medial side, the lateral approach was meaningful in RA cases.
